# Antifungal Activities of Biogenic Silver Nanoparticles Mediated by Marine Algae: In Vitro and In Vivo Insights of Coating Tomato Fruit to Protect against *Penicillium italicum* Blue Mold

**DOI:** 10.3390/md22050225

**Published:** 2024-05-16

**Authors:** Ragaa A. Hamouda, Fatimah Q. Almaghrabi, Ohoud M. Alharbi, Abla D. M. Al-Harbi, Rahaf M. Alsulami, Abrar M. Alhumairi

**Affiliations:** 1 Department of Biology, College of Sciences and Arts Khulais, University of Jeddah, Jeddah 21959, Saudi Arabia; falmaghrabi1@gmail.com (F.Q.A.); ohoudmh32@gmail.com (O.M.A.); 2200456@uj.edu.sa (A.D.M.A.-H.); 2200724@uj.edu.sa (R.M.A.); amhumairi@uj.edu.sa (A.M.A.); 2 Microbial Biotechnology Department, Genetic Engineering and Biotechnology Research Institute, University of Sadat City, Sadat City 32897, Egypt

**Keywords:** silver nanoparticles, pathogenic fungi, *Turbinaria turbinata*, *Solanum lycopersicum* L.

## Abstract

In an attempt to reduce such decay induced by pathogenic causes, several studies investigated the effectiveness of nanoparticles (NPs) that play a vital role in saving food products, especially fruits. Current research delves into biogenic silver nanoparticles (using marine alga *Turbinaria turbinata* (Tt/Ag-NPs) and their characterization using FT-IR, TEM, EDS, and zeta potential. Some pathogenic fungi, which cause fruit spoilage, were isolated. We studied the impact of using Tt/Ag-NPs to protect against isolated fungi in vitro, and the influence of Tt/Ag-NPs as a coating of tomato fruit to protect against blue mold caused by *Penicillium italicum* (OR770486) over 17 days of storage time. Five treatments were examined: T1, healthy fruits were used as the positive control; T2, healthy fruits sprayed with Tt/Ag-NPs; T3, fruits infected with *P. italicum* followed by coating with Tt/Ag-NPs (pre-coating); T4, fruits coated with Tt/Ag-NPs followed by infection by *P. italicum* (post-coating); and T5, the negative control, fruits infected by *P. italicum*. The results displayed that Tt/Ag-NPs are crystalline, spherical in shape, with size ranges between 14.5 and 39.85 nm, and negative charges. Different concentrations of Tt/Ag-NPs possessed antifungal activities against *Botrytis cinerea*, *Rhodotorula mucilaginosa*, *Penicillium expansum*, *Alternaria alternate*, and *Stemphylium vesicarium*. After two days of tomatoes being infected with *P. italicum*, 55% of the fruits were spoilage. The tomato fruit coated with Tt/Ag-NPs delayed weight loss, increased titratable acidity (TA%), antioxidant%, and polyphenol contents, and decreased pH and total soluble solids (TSSs). There were no significant results between pre-coating and post-coating except in phenol contents increased in pre-coating. A particular focus is placed on the novel and promising approach of utilizing nanoparticles to combat foodborne pathogens and preserve commodities, with a spotlight on the application of nanoparticles in safeguarding tomatoes from decay.

## 1. Introduction

Tomatoes (*Lycopersicum esculentum* L.) are a great provider of numerous nutrients, have vital secondary metabolites for human well-being, and contain numerous antioxidants that are abundant in fruits and are essential to human metabolism. Tomatoes are a vital source of bioactive compounds, which have antiproliferative, antioxidant, antimutagenic, anti-atherogenic, and anti-inflammatory properties [1]. Tomato consumption is directly associated with the inhibition of different chronic diseases and carcinogenesis, due to the fact that tomatoes contain lycopene, glutamic acid, beta-carotene, and aspartic acid [2]. Pathogenic fungi caused tomato diseases such as gray and blight leaf mold, and the production of tomatoes suffered a significant loss. The fungi belong to the following families, *Alternaria alternate*, Cladosporium, and Fusarium; these pathogenic fungi have caused severe diseases in tomatoes with high incidence rates in fields [3]. 

*Botrytis cinerea*, *Penicillium italicum*, and *Penicillium digitatum* are known as the most critical post-harvest pathogens [4]. *Penicillium italicum* is the fungi that causes the blue mold disease, which is presently managed by toxic chemicals that are dangerous to the environment and human health. Moreover, *P. italicum* has grown extremely resistant to chemical pesticides as a result of their extensive use [5].

Different microbial agents have been tested against various fungal pathogens to study their effect on the decay of fruits; the application of antagonistic yeasts as bio-control agents resulted in a reduction in protein and showed significant efficacy in inhibiting the decay of peach fruits by oxidative damage. Antagonistic yeasts induced an antioxidant response as a defense mechanism against fungal pathogens, which diminished the oxidative attacks caused by pathogens on peach fruits [6]. A comparison of the inhibitory efficacy of microbial agents on the growth of fungal pathogens has been studied. For example, Meng et al. [7] reported the difference in the mechanisms of Chitosan and Oligochitosan in their inhibitory effects on phytopathogenic fungi and on decay control. Both treatments resulted in controlling the decay of peach fruits and reducing the growth of pathogenic fungi. To clarify, treatment using Chitosan significantly enhanced the activity of peroxidase in pear fruit and showed more control efficiency on postharvest disease than Oligochitosan, which enhanced the activities of chitinase and b-1,3-glucanase.

The use of nanoparticles in postharvest treatments foster antibacterial and antifungal properties in food packaging, nanotechnology, and enhance the mineralization of postharvest fruits [8]. Nanotechnology can be used to enhance the shelf life of fruits and vegetables, because it has yet to be connected to any significant side effects [9].

Nanotechnology seems to manifest promising methods for maintaining food safety and preserving food quality. Coating Chitosan nanoparticles with essential oil proved to have significant potential in extending the shelf life of fruits and improving food quality. The utilization of silver nanoparticles in preparing A-AgNP films proved efficient in promoting the shelf life of fruits and demonstrating antimicrobial activity, which protects fruits against microbial attacks [10]. According to Incoronato et al. [11], using silver nanoparticles in shelf-life tests has resulted in the increased shelf life of packaged Fior di Latte cheese samples. Hence, silver nanoparticles have a proven capability of inhibiting the growth of microorganisms, resulting in prolonged shelf life, diminished deterioration, and maintained food quality with no impact on the dairy microbiota and sensory characteristics of the samples. Silver nanoparticles (AgNPs) positively impacted the incidence and severity of gray mold disease in tomato fruits caused by *Botrytis cinerea* [12]. Silver nanoparticles form a tight coating on the tomato fruit, reducing its rate of transpiration to the surrounding environment. Silver nanoparticles restrict microbial activity, preserving the firmness and color of the fruit [13]. Nanotechnology will hold a great future in the coming years for the shelflife extension of tomatoes [14]. The use of copper nanoparticles during tomato growth alters the accumulation and degradation patterns of bioactive chemicals in postharvest fruits. Copper nanoparticles promote the accumulation of beneficial substances, including total phenols, β-carotene, and vitamin C [15].

Palladium-promoted nano zeolite 5% may be considered the most efficient in preserving the quality of tomatoes and extending their shelf life, which reflects positively on the firmness of the tomatoes and their weight loss, while UV-C 15 min is effective in reducing the severity of decay and maintaining the quality of tomatoes postharvest [16]. It is also notable that Zinc Oxide nanoparticles’ application might have significant potential to improve the nutrient contents of tomatoes and inhibit the growth of microbial agents, hence promoting lycopene content and diminishing tomato spoilage, enhancing the dehydration of tomatoes, and improving their color maturation [8]. The application of Selenium nanoparticles manifested more benefits and fewer risks than Sodium selenate, while the utilization of Selenium nanoparticles induced fruit growth, the accumulation of biomass, improved fruit quality, extended shelf life, improved flowering time, and enhanced yield much more than Sodium selenate, [17]. Similarly, Selenium nanoparticles were biosynthesized using an aqueous extract of Fenugreek seeds in an attempt to combat the infection of postharvest tomatoes by Fusarium. Further, treating tomatoes with Selenium nanoparticles proved effective in thoroughly reducing the infection and maintaining fresh-looking fruits [18]. 

Bioactive compounds derived from algae can help to synthesize nanoparticles and act as reducing, stabilizing, and coating agents, providing a robust coating on nanoparticles [19]. Silver nanoparticles synthesized by marine algae showed antibacterial and anticancer properties, and induced hepatoprotective effects in normal cells against C CL_4_ [20]. Silver nanoparticles bio-fabricated by marine alga *Ulva lactuca* possessed antibacterial activities against some pathogenic bacteria [21]. 

*Ulva fasciata*-based bio-nanoparticles of Ag-NPs inhibited the growth of *Xanthomonas campestris* pv. malvacearum [22]. The silver nanoparticles that were synthesized through an aqueous extract of the marine alga *Turbinaria conoides* showed antimicrofouling agent activity [23]. *Sargassum fluitans* AgNPs showed 79% growth inhibition, which is the highest percentage among all tested strains [24]. Silver NPs have become a central focus due to their effective antimicrobial attributes and minimum toxicity [25]. 

Tomato fruits are the second most important fruit or vegetable crop next to potatoes, so this study aims to utilize biosynthesized silver nanoparticles derived from the marine alga *Turbinaria turbinata* as an antifungal agent against some pathogenic fungi in vitro. We investigate the impact of silver nanoparticles derived from the marine alga *Turbinaria turbinata* as a coating for tomatoes to enhance their storage time and prolong their shelf life. 

## 2. Results and Discussion

### 2.1. FT-IR Analysis of Marine Alga Turbinaria Turbinata

**Figure 1:**
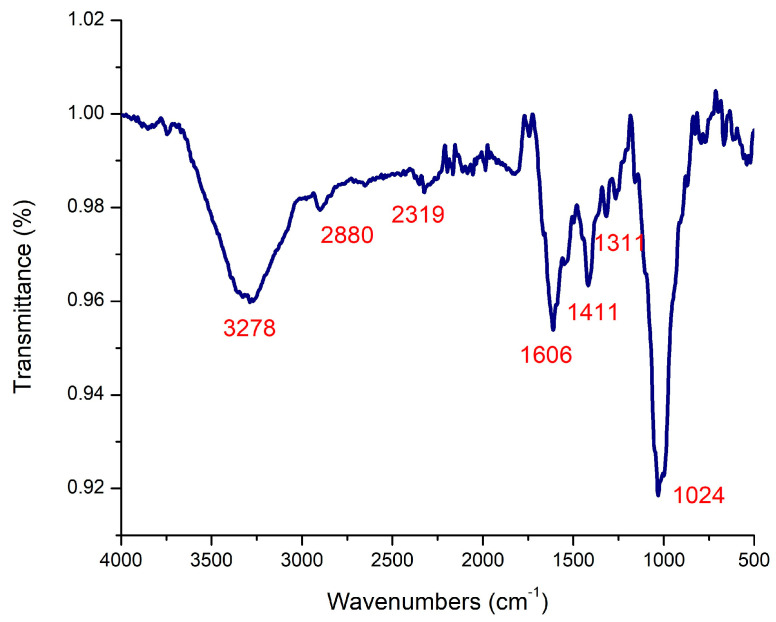
FT-IR analysis of active compounds derived from marine alga *Turbinaria turbinata*.

FT-IR measurements were carried out for the identification of the active compounds found in *T. turbinata marine alga* (Figure 1). The bands observed at the wavenumber 3278 cm^−1^ indicated the presence of amide I. The band at 2880 cm^−1^ represents CH symmetric stretching in CH3 [26]. That at 2319 cm^−1^ is due to the presence of CN stretching [27]. The peak at 1606 cm ^−1^ indicated the presence of hydrogen bonding with neighboring amino acids [28]. A peak at 1411 cm^−1^ related to COO- stretching and C-H bending [29]. The CN stretching vibration is distinct at 1311 cm^−1^ [30]. The band at 1024 cm^−1^ is attributed to C-O stretching [29]. These active groups can reduce silver ions to atoms and subsequently grow to silver nanoparticles [31]. The FT-IR confirms the presence of bio-components in the brown algae extract, which was responsible for the nanoparticles’ synthesis [32].

### 2.2. Nanoparticles’ Characterization

**Figure 2:**
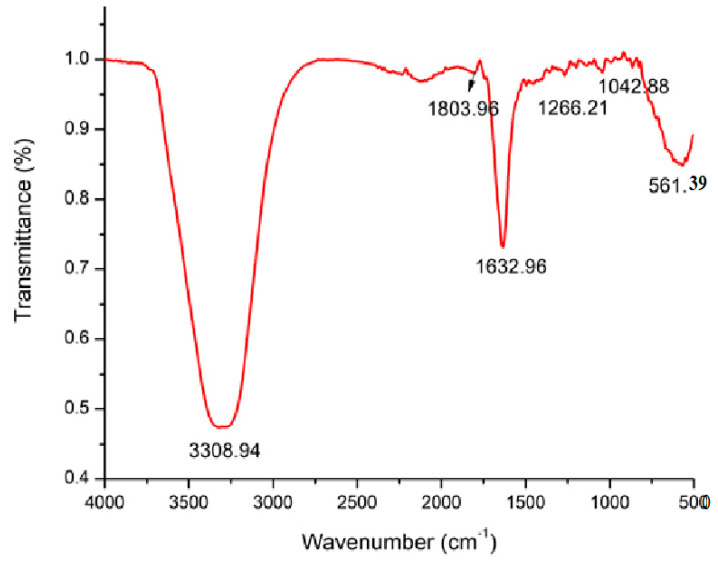
FT-IR analysis of Tt/Ag-NPs derived from marine alga *Turbinaria turbinata*.

FT-IR measurements were carried out for the identification of the biomolecules for the capping and efficient stabilization of the metal NPs synthesized by the marine alga *T. turbinata*. FT-IR spectroscopy shows the presence of different functional groups (Figure 2). The strong and broad band observed at 3308.94 cm^−1^ indicated the presence of the polyphenolic O-H group [33]. The 1632.96 band is due to the stretching vibration of the carbonyl group characteristic of the secondary amides and other compounds containing C=O groups [34]. The peak at 1266.21 cm^−1^ indicated the presence of C-N (strongly), C-H alcohols, carboxylic acids, esters, ethers, alkyl halides, and aromatic amines. The peak at 1042 cm^−1^ denotes the C-H group [33]. Sivakumar et al. [35] reported similar observations in their experiment with the brown alga *T. conoides*. The obtained functional groups attached with Tt-AgNPS could be responsible for stabilizing and reducing agents used in bio-fabricated NPs [36].

**Figure 3:**
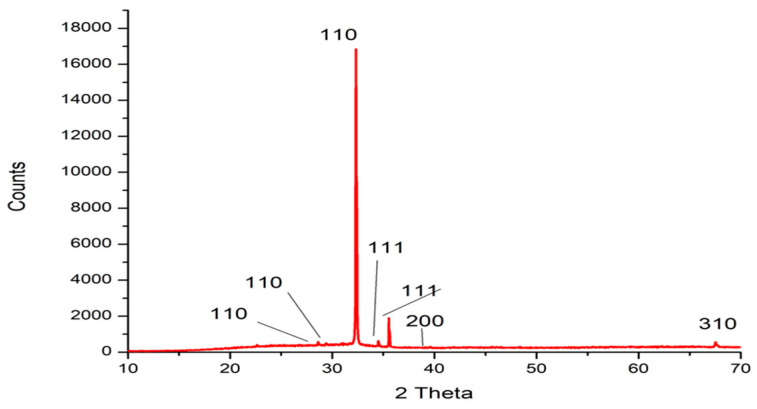
XRD patterns of Tt-AgNPS derived from marine alga *T. turbinata*; the intense peak at 32.321° represents preferential growth in the (110) direction.

The XRD was used to confirm the structural information of nanoparticles, such as size and crystallization. The XRD spectrum (Figure 3 and Table 1) showed seven distinct diffraction peaks at 28.615°, 29.372°, 32.321°, 34.507, 35.544, 39.544, and 67.517°, which are indexed at (110), (110), (110), (111), (111), (200), and (310) of the cubic face-centered silver. The diffraction peaks of silver nanoparticles synthesized by the marine alga *Ulva lactuca* were obtained at 2 Theta 19.843, 29.841, 31.972, and 40.6, which reflect the miller index at 100, 110, 110, and 111, respectively [21]. The diffraction peaks of silver nanoparticles bio-fabricated by *Turbinaria turbinata* were obtained at 2 Theta 27.66°, 32.10°, 46.0°, 54.826°, 57.484°, 67.462°, 74.473°, and 76.750° [37].

**Figure 4:**
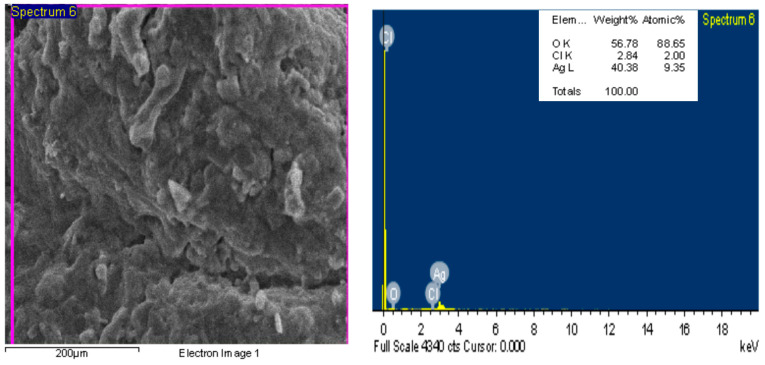
Energy-dispersive X-ray (EDX) of Tt-AgNPS derived from marine alga *T. turbinata*.

Energy-dispersive X-raying (EDX) is an important analytical method used to confirm the sample’s elemental structure. Figure 3 represents the energy-dispersive X-ray (EDX) of Tt-AgNPS. The energy-dispersive X-ray (EDX) clears the presence of the Ag, Cl, and O by weight: 40.38, 2.84, and 56.78. Figure 4 demonstrates the Ag peak located at 3 KeV. The EDX peaks’ silver nanoparticles were synthesized by wet chemical observed at 3.0, 3.2, and 3.4 keV [38]. The EDX analysis established that the silver nanoparticles’ surface plasmon resonance at 2.8 to 3.2 keV was approved for the reduction of silver ions (Ag+ to Ag^0^) [39]. Silver nanocrystals have exhibited an optical absorption peak around 3 keV due to the surface plasmon resonance [40].

**Figure 5:**
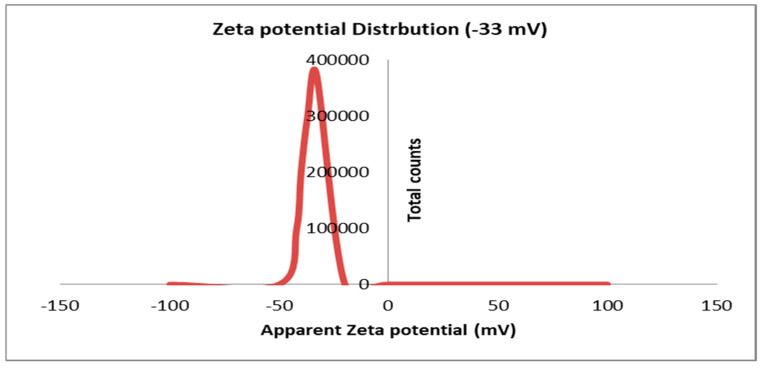
Zeta potential of Tt-AgNPS derived from marine alga *Turbinaria turbinata*.

Figure 5 displays the zeta potential of Tt-AgNPS derived from marine alga *T. turbinata.* The results show the negative zeta potential value (−33 mV) of Tt-AgNPS. The results demonstrate that the high negative charges proved the good stability of Tt-AgNPS due to phytochemical compounds of the brown alga *T. turbinata* that act as capping agents and prevent nanoparticle aggregation [41]. The zeta potential of nanoparticles with values more positive than +30 mV or more negative than −30 mV is reflected as being stable [42]. The negative charge of silver nanoparticles is due to the net charges around the nanoparticles, which come from bioactive compounds from the algal extract, such as the polyphenols compounds on Ag nanoparticles’ surface [43]. A high zeta potential produces potent repulsive force among the particles, thus inhibiting the nanoparticles’ aggregation [44].

**Figure 6:**
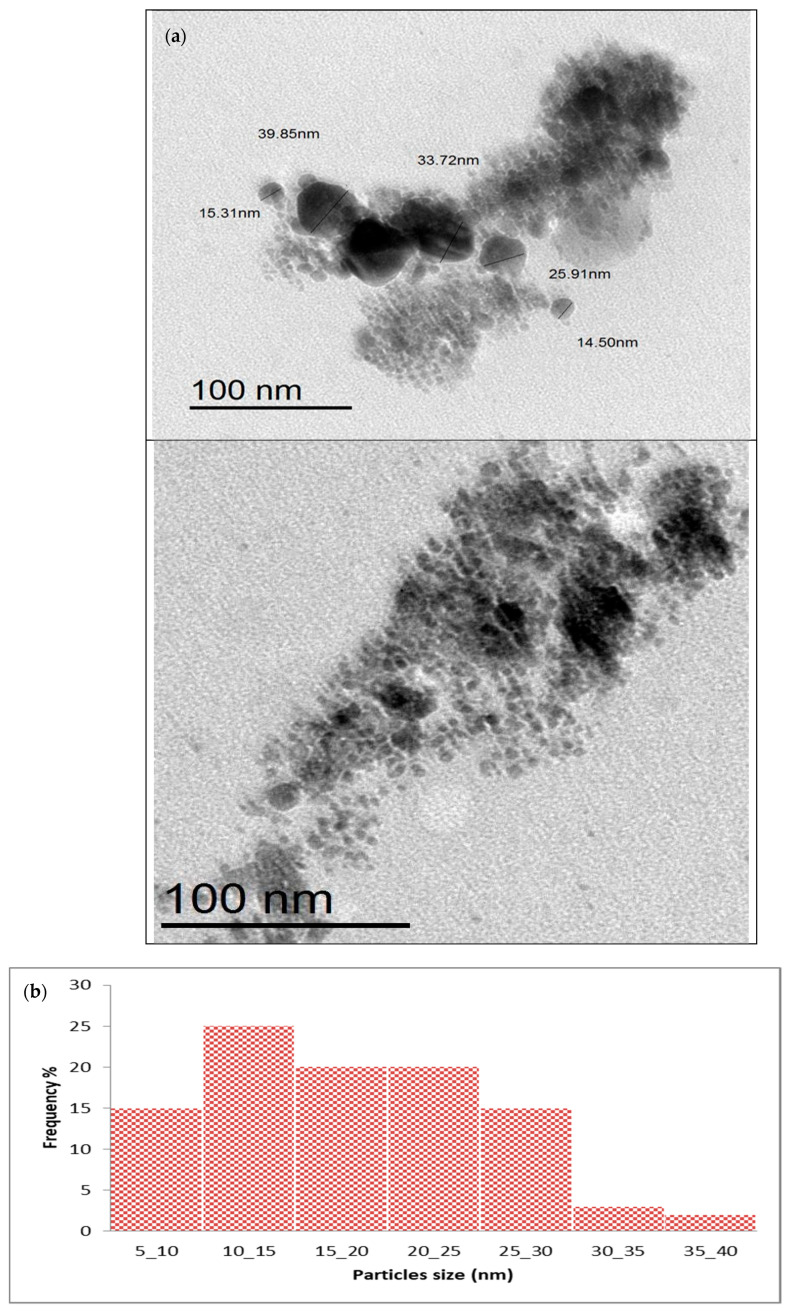
(**a**) TEM image of Tt-AgNPs derived from marine alga *T. turbinata*. (**b**) Particle size distribution of Tt-AgNPs derived from marine alga *T. turbinata*.

Transmission electron microscopy (TEM) shows the morphology and size of Tt/Ag-NPs. The results in Figure 6a display the sizes of Tt-AgNPs, ranging from 14.50 to 39.85 nm, and their spherical shape. It is evident that there is variation in particle size distribution. As shown in Figure 6b, it is evident that the particle size ranged from 5 to 40 nm, and the most predominant nanoparticles are in the range of 10 to 15 nm with a frequency of 25%. The same results were obtained with nanoparticles made biogenic by brown algae. The size of silver nanoparticles bio-fabricated by the marine brown alga *Turbinaria ornata* extract ranges from 64.67 to 81.28 nm [45]. Periaswamy Sivagnanam [46] reported that the silver nanoparticles synthesized by the brown alga *Saccharina japonica* are spherical in shape and range in size from 10 to 40 nm.

### 2.3. Identification and Phylogenetic Analysis

**Figure 7:**
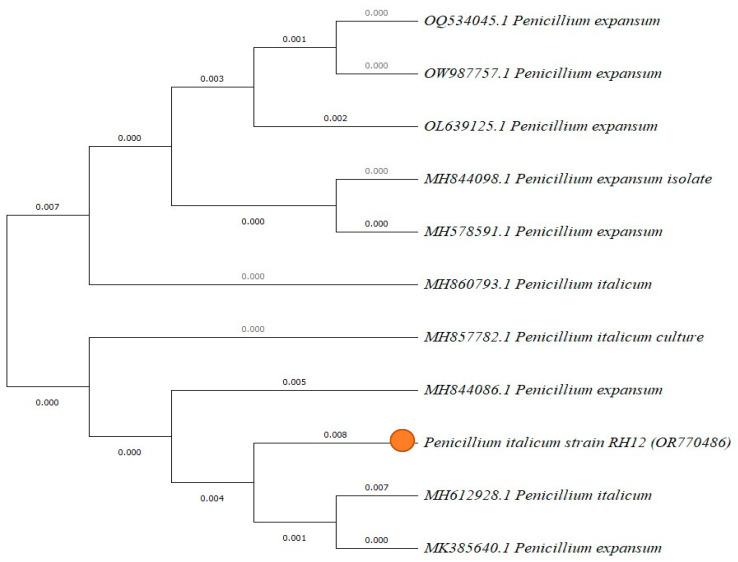
Phylogenetic tree structure built on the Clustral W alignment of ITS sequences of the isolated fungi *Penicillium italicum* RH12 OR770486, with homologue sequences attained from the NCBI GenBank. Orange circle: fungal strain.

Using Blast to associate findings with the National Center for Biotechnology Information (NCBI) databases, the GenBank database was examined for the maximum percentage of identity to the query ITS sequence of the isolated strain. The fungal strains identified according to NCBI are *Botrytis cinerea* RH1 OR770482, *Rhodotorula mucilaginosa* RH5 OR770483, *Penicillium expansum* RH8 OR770484, *Alternaria alternata* RH10 OR770485, *Penicillium italicum* RH12 OR770486, and *Stemphylium vesicarium* RH15 OR770487. Figure 7 demonstrates the phylogenetic tree of *Penicillium italicum* RH12 OR770486, structured and built on the Clustral W alignment of ITS sequences of the isolated fungi, with homologue sequences attained from the NCBI Gen Bank. The *Penicillium italicum* RH12 OR770486 was closed to *Penicillium italicum* MH612928.1 and *Penicillium exapansum* Mk385640.1.

### 2.4. Pathogenicity Tests 

**Figure 8:**
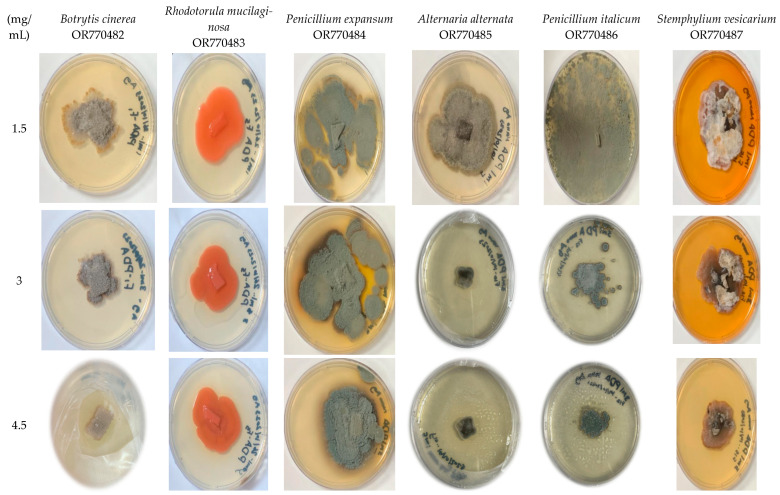
Images of antifungal activates of Tt/Ag-NPs derived from marine alga *T. turbinata*.

As shown in Table 1 and Figure 8, the inhibition of biosynthesized Tt/Ag-NPs was studied against six fungal strains. The synthesized Tt/Ag-NPs were mostly effective in controlling plant pathogenic fungi. All the extracts exhibited different degrees of antifungal activities against *B. cinerea*, *R. mucilaginosa*, *P. expansum*, *A. alternata*, *P. italicum*, and *S. vesicarium*. The current results revealed that the percentage of fungi growth inhibition at the higher concentration (4.5 μg/mL) varied from 20% for *S. vesicarium* to 75.0% for *B. cinerea*. This finding was also reported by Derbalah et al. [47], where *Cassia fistula* AgNPs showed 75.22% against *B. cinerea* at the concentration of 300 ppm. These findings indicated that there are variations in tolerance to Tt/Ag-NPs between different tested fungi. The percentage of inhibition of *B. cinerea* was significantly the highest (75.0%) of other tested fungi at 4.5 mg/mL, followed by *P. italicum*, the inhibition of which was recorded 68.75% at the same concentration. Tt/Ag-NPs also proved to be effective and gave the most promising antifungal activity at a low concentration of 100 µg/mL against *B. cinerea*, *R. mucilaginosa*, *P. expansum*, and *A. alternata*, with inhibitions 66.66, 14.29, 58.33, and 42.86%, respectively (Table 2). The results were broadly in line with that of Ali et al. [48], where *Stemphylium solani* showed similar inhibition percentages of 27% and 21% with *Azadirachta indica*-mediated ZnO-NPs and Chem-ZnO-NPs, respectively, at the concentration of 20 mg. For *A. alternata*, the inhibition percentages shown at the concentration of 40 mg were 45% and 31% for *Azadirachta indica*-mediated ZnO-NPs and Chem ZnO-NPs, respectively. *Turbinaria ornata* AgNPs formed inhibition zones of 13 and 14 mm against *A. alternata* and *P. italicum*, respectively [49]. Kim et al. [50] used different nano-sized silver colloidal solutions against fungal plant pathogens; they found that treatment with a 100 ppm concentration resulted in greater than 90% inhibition against *Stemphylium lycopersici*, *Cladosporium cucumerinum*, *Didymella bryoniae*, *Monosporascus cannonballus*, and *Glomerella cingulata*. Interestingly, 100 μg/mL of Tt/Ag-NPs did not affect *P. italicum* and *S. vesicarium*, and the presence of Tt/Ag-NPs was insufficient to kill the strains. Generally, there was a tendency towards an increase in the zone of inhibition percentage proportional to the increase in the tested concentrations of Tt/Ag-NPs, except for *P. expansum* and *A. alternata*, which show the same percentages in all different concentrations. There were significant differences among all the tested Tt/Ag-NPs concentrations. The inhibition zone showed that the tested fungal strains were susceptible to Tt/Ag-NPs. Our study proved that the silver nanoparticles synthesized from *T. turbinata* could be an effective and promising antifungal agent against the plant pathogenic fungi. The inhibition of fungi by Ag-NPs may be due to the Ag-NPs penetrating the fungal cell membrane, and the leakage of various components such as DNA and proteins outside the fungal cell [51]. The brown algal-mediated silver nanoparticles show increased antifungal activity against pathogenic fungi such as *Aspergillus fumigatus*, *Candida albicans*, and *Fusarium* sp. *S. longifolium* [32].

All fungal strains had the ability to infect the tomatoes within one week. The fungal pathogens, *P. expansum* and *P. italicum*, had a more rapid growth rate compared to other strains after the infection and caused 100% of tomato pathogenicity within only 2 days (Table 3). This rapid infection could be correlated with the high water content in tomatoes, making it more susceptible to spoilage by fungi. Moreover, tomatoes have a pH of 4.2 to 4.5, making them prone to rotting caused by *Penicillium* [52]. Out of 29 tomato samples tested, 79.3% were contaminated with eight species of the genus *Penicillium* [53]. *P. expansum* caused pectin depolymerization in tomato cell walls due to the action of pectinases, which may increase the porosity of the cell wall, allowing fungus colonization and facilitating the progress of fungal infection [54]. On the other hand, *B. cinerea* and *S. vesicarium* recorded a slow growth in the first two days of inoculation until the fourth day. *A. alternata* infected 20, 96, and 100% of tomatoes on the 2nd, 4th and 7th day, respectively. Kalyoncu et al. [55] reported the presence of *A. alternata* and *Fusarium oxysporum* in the spoilt tomato fruits. *R. mucilaginosa* had the lowest infection, with 16% in the first two days.

### 2.5. Determination of Physical and Chemical Characteristics 

**Figure 9:**
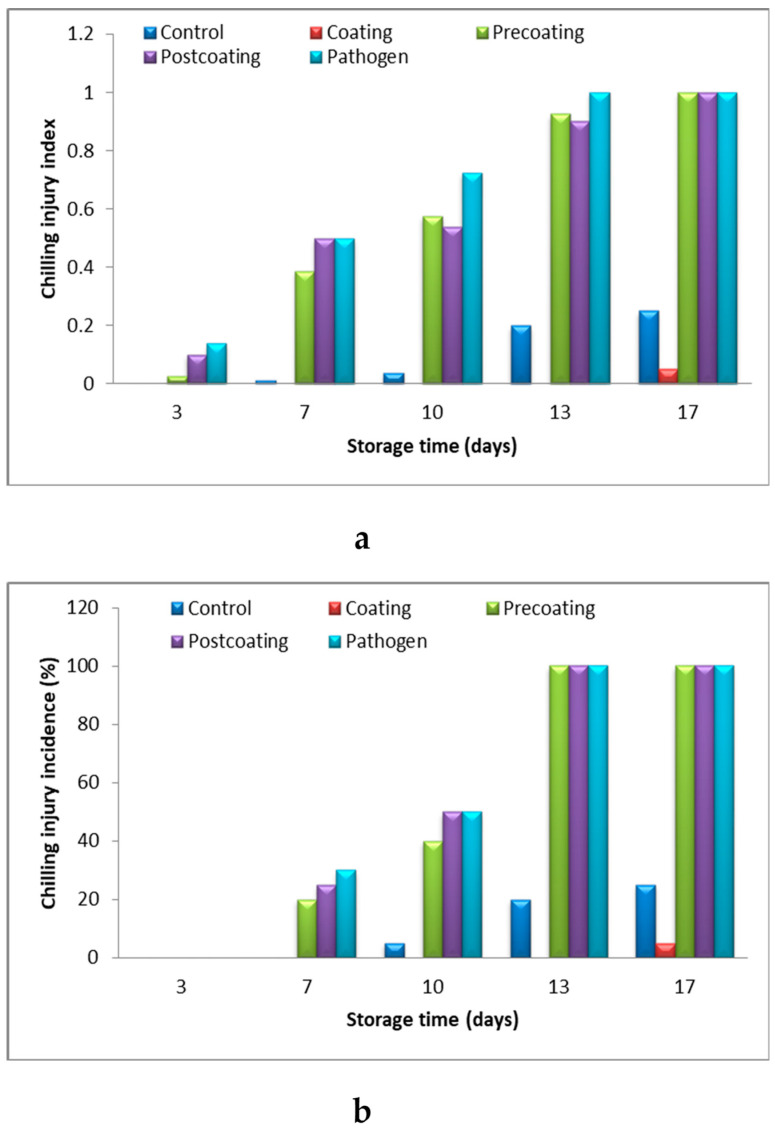
Change in (**a**) chilling injury index, and (**b**) chilling injury incidence %, of postharvest tomato coated with Tt-AgNPs derived from marine alga *T. turbinata* during storage periods at ambient temperatures. (Coating: sprayed with nanoparticles; pre-coating: infected, followed by spraying with Tt-AgNPs; and post-coating: spraying with Tt-AgNPs, followed by infection).

The data in Figure 9a show a higher chilling injury index (CII) in the pathogen group (infected tomato fruits) compared to other treatments, except after 17 days of storage, where the control, post-coating, and pathogen groups have the same CII. Fruits coated with Tt-AgNPs exhibited the lowest rate of chilling injury compared to other treatments. Coating treatments were found to effectively reduce the chilling injury. The cold stress on cherry tomatoes uncoupled key physiological, biochemical, and molecular processes that occur during the normal progression of storage and ripening [56]. 

**Figure 10:**
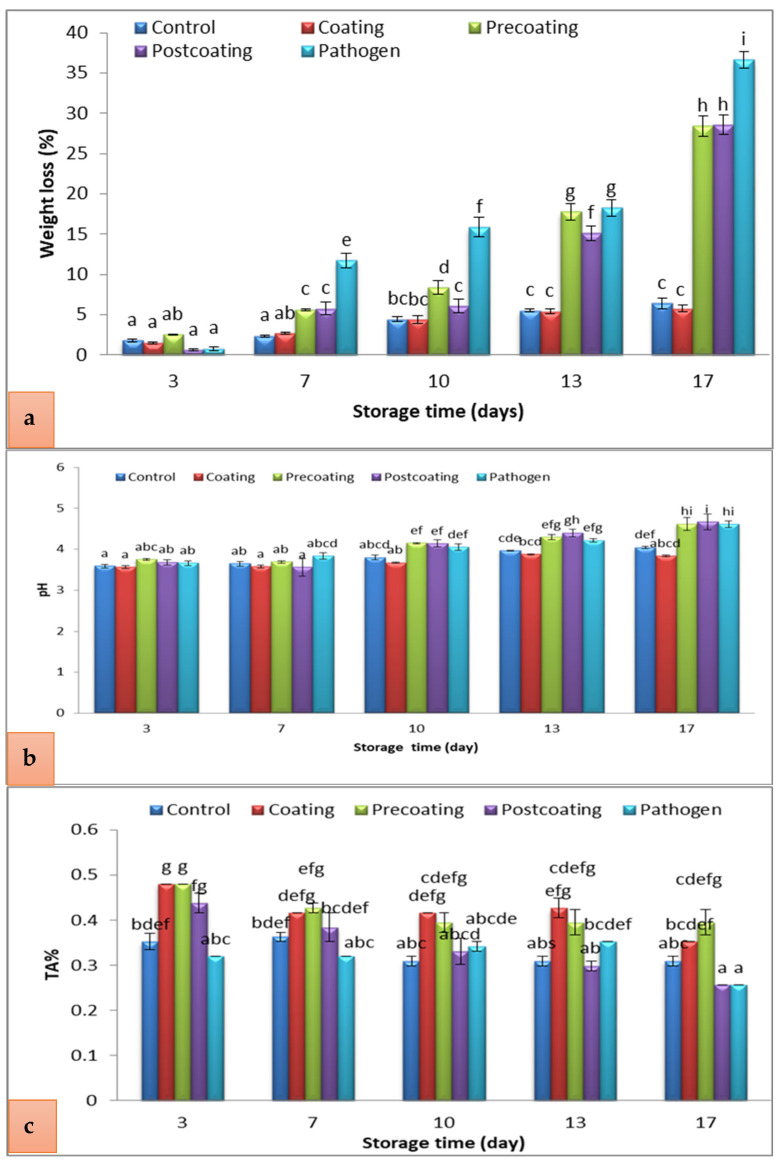
Change in (**a**) weight loss (%), (**b**) pH, and (**c**) TA (%) of postharvest tomato, coated with Tt-AgNPs derived from marine alga *T. turbinata*, during storage periods at ambient temperatures. Values with the same letter are not significantly different (*p* < 0.05). (Coating: sprayed with nanoparticles; pre-coating: infected, followed by spraying with Tt-AgNPs; and post-coating: spraying with Tt-AgNPs, followed by infection). Bars represent error bars.

As the storage time advanced, the fruit gradually lost weight. The highest weight loss percentage was recorded after 17 days in the pathogen group (35%), followed by the 30% of both post-coating and pre-coating groups, while the lowest was after 3 days of treatment in post-coating fruits (Figure 10a). The higher weight loss in the pathogen group (infected tomato fruits) was caused by higher respiration rates, ethylene production, dehydration, and metabolic activity [57,58]. This water loss is mainly associated with the fruit’s respiration, which produces water as a final product, in which the increased rate of respiration results in more water loss in the control fruit [59]. The polyphenol-grafted Chitosan Ag-NP coating prevented the weight loss of tomato fruits during storage due to the linkage between polyphenols and Chitosan that created a tight structure as an effective barrier, limiting the transpiration process of the tomatoes [60].

Figure 10b shows the pH increase during storage time in all treatments and controls. By storage day 17, the post-coating tomatoes had a higher pH than other treatments. Nevertheless, the pathogen and pre-coating fruits’ pH levels did not differ from each other. The pH was approximately within the normal range and ranged from 3.7 to 4.5. The treatment of coated tomatoes maintained a lower fruit pH among other treatments’ pH levels (3.8) after 17 days of storage. The maximum pH value of healthy tomato fruit coated with silver nanoparticles among the treated fruits was (3.3) during storage periods [12]. The pH of all postharvest tomatoes coated with Cassava Starch and the control was increased during storage [61]. It was found that the TA% ranged between 0.26 and 0.47% (Figure 10c). The coating slightly decreased the titratable acidity % of the tomatoes. However, as the storage days progressed, the TA % of tomatoes reduced significantly. A significant decrease was observed in acidity values and pH in aloe vera gel-coated tomatoes with a similar pH ratio, which increased as acidity decreased [62].

**Figure 11:**
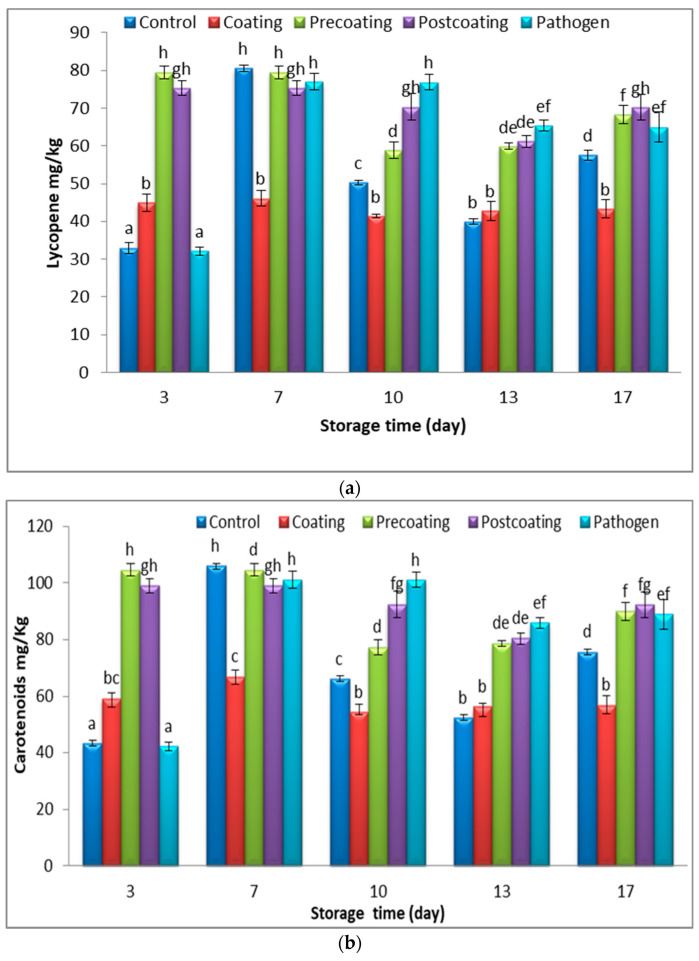

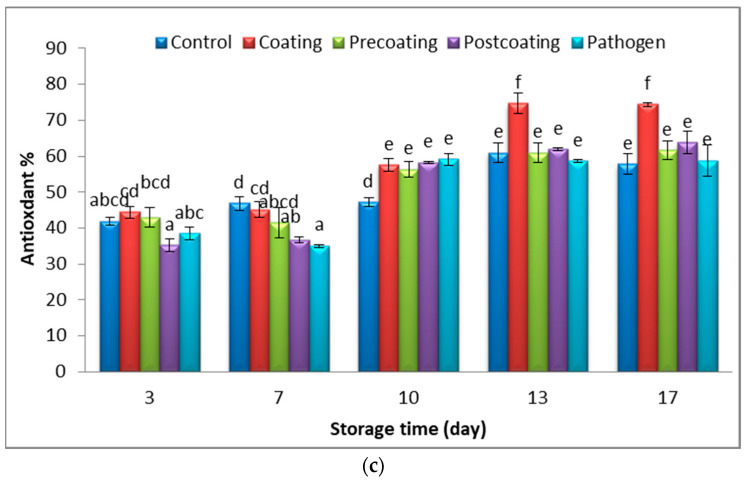
Change in (**a**) lycopene (mg/Kg), (**b**) carotenoids (mg/Kg), and (**c**) antioxidant % of postharvest tomato, coated with Tt-AgNPs derived from marine alga *T. turbinata*, during storage time at ambient temperatures. Values with the same letter are not significantly different (*p* < 0.05). (Coating: sprayed with nanoparticles; pre-coating: infected, followed by spraying with Tt-AgNPs; and post-coating: sprayed with Tt-AgNPs, followed by infection). Bars represent error bars.

It was found that the carotenoid content in the healthy tomatoes coated with Tt-AgNPs was nearly 60 mg/Kg during the storage period (Figure 11b). The use of coating may not affect the carotenoid synthesis during the storage time. The carotenoids increased in the case of pre-coating-, post-coating-, and pathogen-group tomato fruits in 3 to 10 days. The β-carotene and ascorbic acid content increased in tomatoes infected by *Botrytis cinerea* compared with the control fruit [63]. After ten days, the significant reduction in carotenoids occurred in the pre-coating, post-coating, and pathogen groups may be due to the fungi growing on fruits decomposing organic materials such as carotenoids. Ten fungal strains showed an efficient degradation of β,β-carotene through the discoloration of β,β-carotene [64]. The results demonstrate there is no significant change in lycopene contents in tomato fruits coated with Tt-AgNPs during storage periods; meanwhile, a significant decrease occurred at 13 days of storage in all other treatments. Lycopene is highly reduced under O_2_ and extreme temperatures, although encapsulation may lower these losses while permitting a controlled lycopene release over time [65]. The nano-coatings protect fruits from the entry of oxygen and CO_2_, which alters the atmosphere by producing semi-permeable barriers, thus lessening respiration, water, and oxidation reaction rates [66]. The results showed that the antioxidant percentage of tomato fruits consistently increased for all the treatments at up to 13 days and remained the same on day 17 (Figure 11c). The results demonstrate that the antioxidant activities were increased in healthy coated tomato fruits compared with others treated at 13 and 17 days of storage, and there was no significant change in antioxidant activity after 13 days of storage in healthy coated tomato fruits. The Ag-NPs treatment can maintain postharvest Mangosteen fruits by delaying weight loss, hardening the pericarp, decreasing the TSS/TA ratio, and sustaining the ascorbic acid content [67]. The silver nanoparticles act as efficient preservatives which enhance the antioxidant activity, thereby lessening the harmful impact of accumulated free radicals [68].

**Figure 12:**
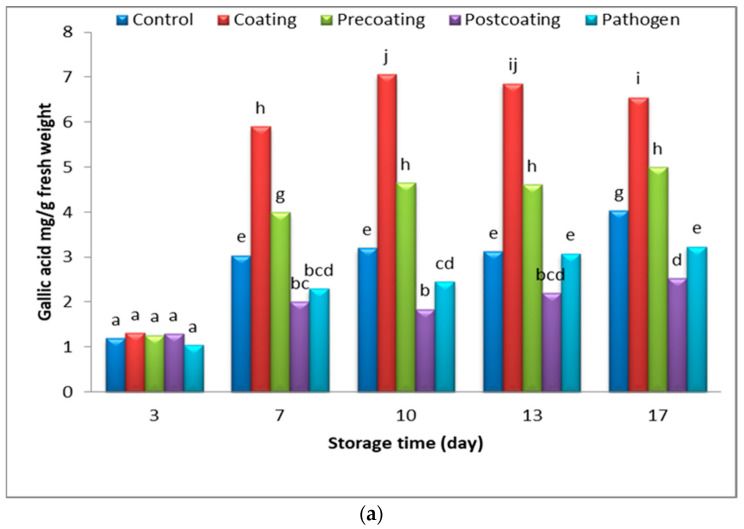

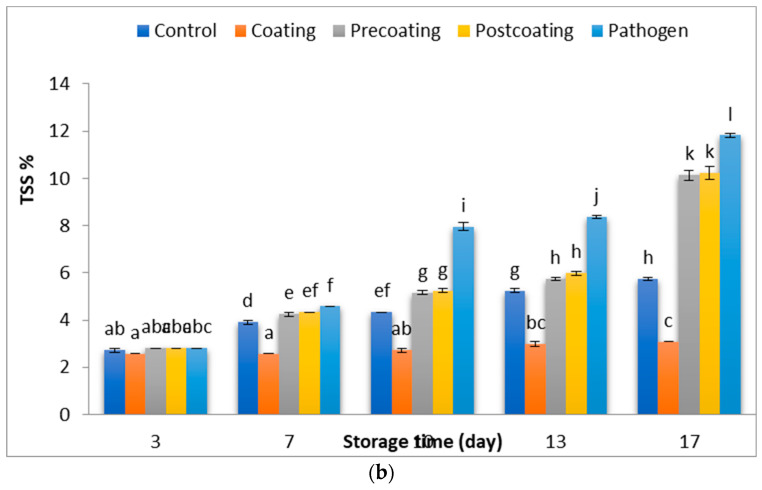
Change in (**a**) phenolic contents (mg/g) and (**b**) TSS% of postharvest tomato, coated with Tt-AgNPs derived from marine alga *T. turbinata*, during storage time at ambient temperatures. Values with the same letter are not significantly different (*p* < 0.05). (Coating: sprayed with nanoparticles; pre-coating: infected, followed by spraying with Tt-AgNPs; post-coating: sprayed with Tt-AgNPs, followed by infection). Bars represent error bars.

The results in Figure 12a demonstrate the significant increase in polyphenol contents in postharvesting tomatoes in all treatments; the elevation of phenolic contents was noticed in silver-coated postharvest tomatoes during storage periods. Non-significant changes in the phenol contents in pre-coating- and pathogenic-group tomato fruits after seven days of storage periods were observed. Meanwhile, a slightly significant increase was observed in the phenolic contents in post-coating tomato fruits. Ali et al. [69] reported the antioxidants and polyphenol contents of loquats (*Eriobotrya japonica* Lindl) increased when coated with silver nanoparticles during 1 month of storage. Longkong fruit coated with both two concentrations of silver particle alginate (0.45 and 0.90 mg·L^−1^) indicated a higher total phenolic content compared to the control fruits [70].

Total soluble solids (TSSs) display a vital role in measuring fruit quality and consumer suitability [71]. The results in Figure 12b demonstrate that the TSSs of the silver nanoparticles coating the tomato fruits slightly increased during the storage period; meanwhile, the TSSs significantly increased in other treatments and the control. Silver nanoparticle-coated apricots retained their TSSs and TA during the storage period and showed minimal weight loss linked to decreased respiration, and reduced respiration rate following AgNP treatment may be due to the fact that silver ions generated by Ag-NPs damaged the respiratory electron transport system and blocked the Krebs cycle’s respiratory activity by effluxing intracellular ions [9]. The increase in TSSs was slightly lower in both fruit coated with guar gum-based Ag-NPs and carboxymethyl cellulose compared to the control [72].

### 2.6. Relationship of Postharvest Tomato Characteristics

**Figure 13:**
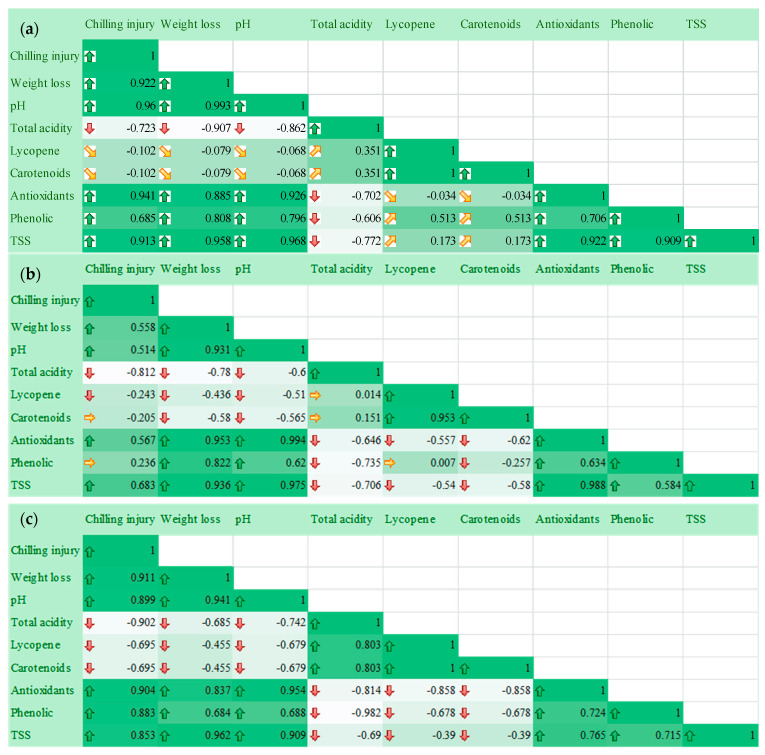

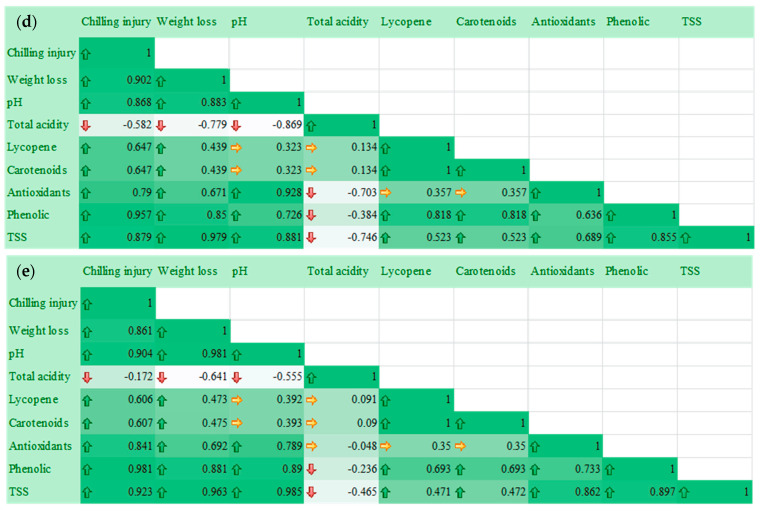
(**a**) Pearson’s correlation coefficient heat map for measured parameters of postharvest tomato during storage times (positive correlations are displayed in green and negative correlations are displayed in white); (**b**) Pearson’s correlation coefficient heat map for measured parameters of postharvest tomatoes coated with Tt-AgNPs during storage times (positive correlations are displayed green and negative correlations are displayed in white); (**c**) Pearson’s correlation coefficient heat map for measured parameters of infected postharvest tomato pre-coated with Tt-AgNPs during storage times (positive correlations are displayed in green and negative correlations are displayed in white); (**d**) Pearson’s correlation coefficient heat map for measured parameters of infected postharvest tomato post-coated with Tt-AgNPs during storage times (positive correlations are displayed in green and negative correlations are displayed in white); (**e**) Pearson’s correlation coefficient heat map for measured parameters of infected postharvest tomato during storage times (positive correlations are displayed in green and negative correlations are displayed in white (upper direction arrows are positive correlation, lower direction negative correlation).

A Pearson’s correlation coefficient test was used to examine the inter-correlation of the tomato fruits’ quality aspects during their storage time. Figure 13a demonstrates the Pearson’s correlation heat map for the measured parameters of postharvest tomatoes during their storage time. Correlations were found among chilling injury, weight loss, pH, total acidity, lycopene, carotenoids, antioxidants, phenolic contents, and TSS. Chilling injury is positively correlated with weight loss (r^2^ = 0.922), pH (r^2^ = 0.96), antioxidants (r^2^ = 0.94), TSS (r^2^ = 0.913), and phenolic contents (r^2^ = 0.685), and negatively correlated with total acidity (r^2^ = −0.723), lycopene (r^2^ = −0.102), and carotenoids (r^2^ = −0.102). The total acidity is negatively correlated with antioxidants, phenolic contents and TSS. Figure 13b displays the Pearson’s correlation coefficient heat map for the measured parameters of postharvest tomatoes coated with Tt-AgNPs during their storage time. The weight loss is strongly correlated with pH (r^2^ = 0.93), antioxidants (r^2^ = 0.93), phenolic contents (r^2^ = 0.822), and TSS (r^2^ = 0.936). The antioxidant level of the coating of tomato fruits is positively correlated with phenolic contents (r^2^ = 0.634) and TSS (r^2^ = 0.988).

Figure 13c,d demonstrate Pearson’s correlation coefficient heat map for the measured parameters of infected postharvest tomatoes pre-coated and post-coated with Tt-AgNPs during their storage time. There were some different correlation coefficients for the measured parameters in the results of tomatoes pre-coated and post-coated with Tt-AgNPs. In postharvest tomatoes pre-coated and post-coated with Tt-AgNPs, the total acidity is negatively correlated with chilling injury, weight loss, and pH. In the case of postharvest tomatoes pre-coated with Tt-AgNPs, the antioxidant level is negatively correlated with total acidity (r^2^ = −0.814), lycopene (r^2^ = −0.858), carotenoids (r^2^ = −0.858), and each phenolic content, and TSS is negatively correlated with total acidity, lycopene, and carotenoids. Meanwhile, in the case of postharvest tomatoes post-coated with Tt-AgNPs, antioxidants, phenolic contents, and TSS are negatively correlated with total acidity and positively correlated with both lycopene and carotenoids.

**Figure 14:**
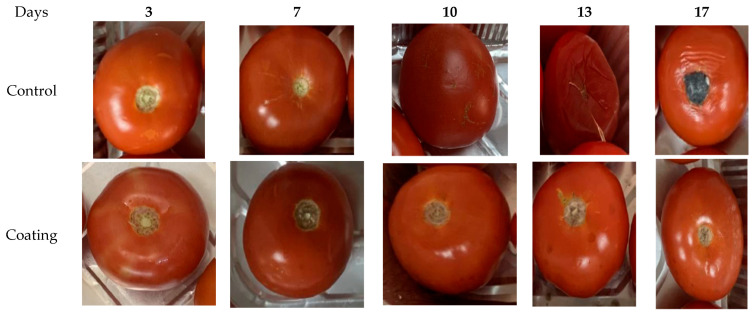

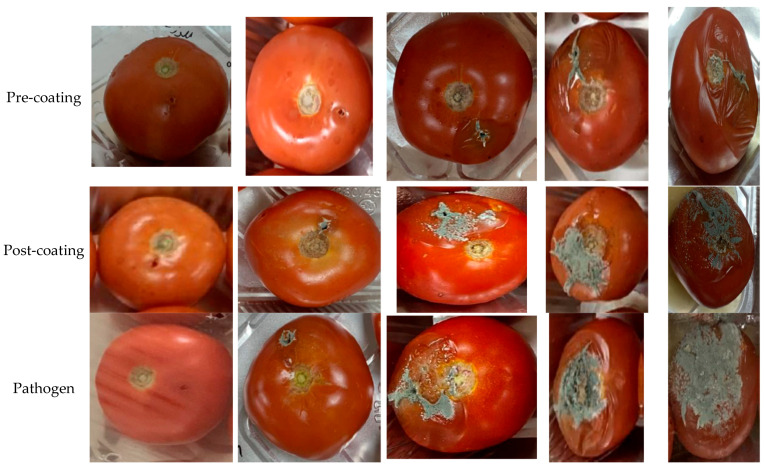
Visual appearance of treated tomato during storage periods at ambient temperatures (coating: sprayed with nanoparticles; pre-coating: infected, followed by spraying with Tt-AgNPs; post-coating: sprayed with Tt-AgNPs, followed by infection).

The results in Figure 13e show Pearson’s correlation coefficient heat map for the measured parameters of infected postharvest tomatoes during the storage time. The total acidity is negatively correlated with chilling injury, weight loss, and pH, and the same results were obtained in the cases of pre- and post-coating groups. The total acidity is negatively correlated with antioxidant level, phenolic content, and TSS; the same results were obtained for post-coating-group tomatoes. The results in Figure 14 demonstrate the visual appearance of treated tomatoes during the storage periods at ambient temperatures. The infection levels of the tomatoes by *Penicillium italicum* increased during the storage period and were higher than the uncoated samples (control). It was noticed that the infection levels of the coated infected tomato declined during the storage period and appeared after 17 days of storage. The coated healthy samples maintained a good appearance for 17 days of storage. Bacteriocins AgNP-coated paper improved the tomatoes’ shelf life by preserving their quality, extending their shelf life, and delaying microbiological decomposition [73].

## 3. Materials and Methods

### 3.1. Preparation of Silver Nanoparticles

#### 3.1.1. Alga Collection

The brown alga *T. turbinata* was collected in December 2022 from the beach of Tabuk, Saudi Arabia, (28°01′54.4″ N 35°12′45.1″ E) and identified according to [74]. The *T. turbinata* was initially washed and then shaded. The alga was carefully cleaned in the laboratory with running tap water, oven-dried until a constant weight was achieved, and ground into powder using an electric blender.

#### 3.1.2. Algal Extract

About 1.0 g of the dried alga *T. turbinata* was added to 100 mL of distilled water. The mixture was then boiled for an hour, cooled, filtered, and then completed to the same volume (100 mL). 

#### 3.1.3. Biosynthesis Silver Nanoparticles (Tt/Ag-NPS)

In total, 1 mM of silver nitrate was added to distilled water (90 mL). At 70 °C, the *T. turbinata* extract (10 mL) was added, drop by drop, to the silver nitrate solution, being stirred until the mixture color turned dark brown. The silver nanoparticles (Tt/Ag-NPS) were centrifuged (12,000 rpm, 15 min), washed three times with D.D water to remove the unreacted particles, and dried at 60 °C [75]. 

#### 3.1.4. Silver Nanoparticles’ Characterization

##### FT-IR

The functional groups of Tt/Ag-NPS were inspected using a Fourier transform infrared spectrometer (FT-IR), Thermo Fisher Nicolet IS10, (Waltham, MA, USA) spectrometer; the FT-IR spectrum ranged between 400 and 4000 cm^−1^. The nanoparticles were incorporated with KBr Pellets to determine the active groups.

##### Transmission Electron Microscopy (TEM)

The shape and size of biosynthesized Tt-AgNPS were assessed using TEM (JEOL JSM-6510/v, Tokyo, Japan). A drop of bio-fabricated silver nanoparticle solution was put on the carbon-coated copper grids and kept overnight under vacuum desiccation. The carbon-coated copper grids were then loaded onto a specimen holder. 

##### XRD Analysis

The crystallite of Tt-AgNPS was evaluated using X-ray powder diffraction (XRD) (PAN Analytical X-Pert PRO, spectris plc, Almelo, The Netherlands). The Tt/Ag-NPS size was inspected using Scherrer’s equation. Crystal size L = λk/c β θ, where λ = 0.1540 nm, k is the constant factor of 0.91, θ = diffraction angle in radians, and β = full width at half maximum (FWHM). 

##### Energy-Dispersive Spectroscopy and SEM

A field emission scanning electron microscope equipped with energy-dispersive spectroscopy (EDS) (JEOL JSM-6510/v, Tokyo, Japan) was used to investigate the surface morphology and element contents of Tt/Ag-NPS. Silver nanoparticle solutions were centrifuged for 20 min at 10,000 rpm and drop-coated onto a thin glass film. The samples were then examined using scanning electron microscope compositional analysis, and the conformation of the presence of elemental silver was carried out through energy-dispersive X-ray spectroscopy.

##### Zeta Potential

The zeta potential of the Tt/Ag-NPS solution provided details of the stabilization of the nanoparticles (Malvern zeta size nano-Zs90, Malvern, PA, USA).

### 3.2. Fungi Isolation 

The infected blueberries were cut into small 2–3 mm pieces, and three pieces were positioned on a 2.5% Potato Dextrose Agar (PDA) medium amended with 10 mg L^−1^ of rifampicin and 200 mg L^−1^ of ampicillin. The plates were kept at an ambient temperature (25 °C), for 6 to 10 days. Pure cultures were preserved for future use at 5 °C, and the separated fungi were molecularly identified [76].

#### 3.2.1. Identification and Phylogenetic Tree of Fungi

The fungal strains were identified based on molecular analysis using the internal transcript spacer regions (ITS1 and ITS4). The polymerase chain reaction (PCR) was performed to amplify the ITS region of the fungal isolates using the universal ITS primers, ITS1 (5′-TCCGTAGGTGAACCTGCGG-3′) and ITS4 (5′-TCCTCCGCTTATTGATATGC-3′). All obtained fungal ITS sequences have been deposited in Gen Bank and subjected to BLAST search analysis in the National Center of Biotechnology Information (NCBI) database to compare the sequence homology with closely related species. The phylogenetic tree was assembled in the MEGA11 program [77].

#### 3.2.2. In Vitro Antifungal Activities 

Different concentrations (100, 200, and 300 µg/mL) of silver nanoparticles (1 mL) were blended in a PDA medium. After the solidification of PDA, 5 mm diameter agar discs from 6-day-old cultures of *Botrytis cinerea* OR770482, *Rhodotorula mucilaginosa* OR770483, *Penicillium expansum* OR770484, *Alternaria alternata* OR770485, *Penicillium italicum* OR770486, and *Stemphylium vesicarium* OR770487 were then placed in the center of the PDA-NPs Petri dishes and incubated at (30 °C). After 6 days of incubation, the colony diameter was measured. The percentage inhibition of the radial growth of the fungal and yeast pathogens was assessed according to the following equation [78]:

Growth inhibition (%) = ((RG1 − RG2)/RG1) × 100

where DR1 = radial growth of the fungal pathogen without treatments in the positive control, and DR2 = radial growth of the pathogen on the treated plate. 

#### 3.2.3. Determination of Susceptibility of Tomato Fruits to the Pathogen’s Strains

Twenty-five healthy tomatoes *Solanum lycopersicum* L. were washed with tap water, dipped in distilled water, and surface-sterilized by % 1 NaOCl for 15 min, then washed with sterile water, followed by being left to dry in aerobic dry conditions [18]. A wound (diameter of 1.5 mm and depth of 3 mm), was made in tomato fruits, followed by receiving an injection of 20 µL of 106 conidia/mL *Penicillium italicum* RH12 OR770486 pathogenic fungus suspension. The treated fruits were put in transparent closed polythene bags, and the percentage of spoiled fruits was determined as the number spoiled fruits as a % of the healthy fruits. 

### 3.3. Silver Nanoparticles Used in the Preservation of Tomato

After washing the tomato fruits, they were sterilized using % 1 NaOCl, followed by sterile water, and left to dry; the coating solution was formulated by mixing 1.5% glycerol as a plasticizer and adding Tt-AgNPs to reach a concentration of 1.5 mg/mL. The solution was agitated for 10 min at an ambient temperature using a magnetic stirrer (400 rpm). The tomato fruits were randomly divided into five groups: the first was the control, without any treatments; group 2 (the coating group) included healthy fruits coated with Tt-AgNPs by spraying each fruit with 3 mL of the coating solution; in group 3, infected tomato fruits were sprayed with the coating solution after 2 hrs of infection (pre-coating); in group 4, healthy fruits were sprayed with coating solutions, and after two hours infected with *Penicillium italicum* RH12 OR770486 (post-coating); and group 5 included infected tomato fruits.

#### Chilling Injury Index and Incidence 

Twenty fruits per treatment were used for each quality assessment. Samples from each treatment were assessed individually on the initial day and days 3, 7, 10, 15, and 17 during storage. The overall quality was evaluated on a 1–4 scale matching the percentage of surface area decayed, where 1 = more than 50% of the surface disturbed, 2 = 25 to 50% of the surface disturbed, 3 = 1 to 25% of the surface disturbed, and 4 = excellent (no surface disturbed). The results were conveyed as an overall chilling injury index and calculated using the following equation [79]:

Chilling injury index = ((Σ CI level × number of fruit at the CI level)/(total number of fruits) × 4) × 100

Incidence of chilling injury (%) = ((Number of injured fruits)/(Total fruit number)) × 100

### 3.4. Physical and Chemical Characteristics’ Determination

#### 3.4.1. Weight Loss (%)

The weight of tomato fruits was measured after 3, 7, 10, 13, and 15 days, and we determined the weight loss by using the following equation [79]

Weight loss (%) = ((starting tomato weight-weight of tomato at after storage period)/(starting weight)) × 100

#### 3.4.2. Titratable Acidity (TA) and pH Measurements

The tomato was extracted by homogenization for 2 min at a high speed using a food blender and filtered using a muslin cloth. In total, 2 mL of the obtained juice was added to 38 mL of distilled water. Further, 2 mL of diluted tomato juice were titrated against 0.1 N NaOH, using phenolphthalein as an indicator.

The TA % was obtained by using the following equation [18]:

A% = ((vol∶ NaOH (mL) × 0.1 (normality of NaOH) × 0.064)/(mL of tomato Juice)) × 100

Additionally, 0.064 is the acid milliequivalent factor.

After the filtration of tomato juice, the pH was determined using a pH meter.

#### 3.4.3. Total Lycopene and Carotenoids

We ground one gram of tomato juice in 14 mL hexane and acetone (3:2 *v*/*v*), mixing it well using a magnetic stirrer at 400 rpm for 15 min, then centrifuged it at 100,000 rpm for 10 min at 4 °C. The liquid phase was completed to 25 mL and measured using a spectrophotometer at 503 and 450 nm. The total lycopene and carotenoid pigments were calculated according to the following equations [80,81]:

Total lycopene content (µg/g fr. W) = (Abs (503 nm) × 3.12 × volume of sample × Dilution factor)/(gram of sample)

Total carotenoids content (µg/g fr. W) = ((4.642 × Abs (450) − 3.091 × Abs (503)) × volume of sample × Dilution factor)/(gram of sample)

#### 3.4.4. Tomato Fruit Extraction

Briefly, 0.1 g of tomato sample was extracted with 10 mL methanol using a mortar and then centrifuged at 5000 rpm for 10 min.

#### 3.4.5. Total Phenolic

The extracted sample (0.2 mL) was added to 2.6 mL of deionized water, 2 mL of 7% (*w*/*v*) Na_2_CO_3_, and 0.2 mL of Folin–Ciocalteu’s phenol reagent. The mixture was then incubated at room temperature for 90 min. After that, a spectrophotometer was used to measure the absorbance at 750 nm. The total phenolics content was conveyed as mg of Gallic acid equivalents (GAEs) per gram of fresh weight of the sample.

#### 3.4.6. Determination of Free Radical Scavenging Activity (DPPH)

In total, 0.3 mL of methanol extract was added to 2.7 mL of DPPH 01 M (cold methanol solution) and left in the dark for 60 min at 4 °C. The absorbance was measured at 515 nm.

% Antioxidant activity = ((Abs control − Abs sample)/(Abs control)) × 100

#### 3.4.7. Total Soluble Solids (TSS %) 

The total soluble solids content (TSS %) was calculated by a hand Refractometer [82]. A drop (less than 0.5 mL) of the tomato fruit was added to the Refractometer, and the reading was represented as a percentage of the total soluble solids (°Brix).

### 3.5. Statistical Analysis

The data were analyzed using SPSS software version 16 (one-way, completely randomized ANOVA), and the results were compared using Duncan’s multiple range ≤ 0.05 level of probability. The Pearson correlation coefficient (PCC) measures the linear correlation between two datasets. Correlation analyses were used to correlate the chilling injury, weight loss, pH, total acidity, lycopene, carotenoids, antioxidants, phenolic content, and TSS.

## 4. Conclusions

This study highlights the synthesis of biogenic silver nanoparticles via the marine alga *Turbinaria turbinata* and their applications as antifungal agents in vitro and their use as a coating for postharvest healthy and *Penicillium italicum*-infected tomato fruits. The results demonstrated that the silver nanoparticles are stable, spherical in shape, and crystal, and have a negative charge. The different concentrations of silver nanoparticles possessed antifungal activities against all tested fungi. The silver nanoparticles delayed the increase in TSS, and fruit decay, and increased the shelf life of coated fruits’ health, as well as infected tomatoes after and before infection in comparison to the infected control. Low concentrations of silver nanoparticles create barriers around the fruits, blocking the passage of CO_2_, O_2_, and moisture, and extend their shelf life.

## Figures and Tables

**Table 1:** Simple peaks’ indexing of Tt-AgNPS derived from marine alga *T. turbinata*.

2 Theta°	Intensity	hkl	Crystal Size (nm)
28.615	2.40	110	40.99
29.372	2.20	110	35.70
32.321	100.00	110	34.46
34.507	3.10	111	39.61
35.544	8.50	111	26.06
39.544	0.80	200	28.13
67.517	2.40	310	88.80

**Table 2:** The percentage of antifungal activates of Tt/Ag-NPs derived from marine alga *Turbinaria turbinata*.

Fungi	Concentration (mg/mL)		
	1.5	3	4.5
*Botrytis cinerea* (OR770482)	66.66	66.66	75.0
*Rhodotorula mucilaginosa* (OR770483)	14.29	42.58	42.58
*Penicillium expansum* (OR770484)	58.33	58.33	58.33
*Alternaria alternata* (OR770485)	42.86	42.86	42.86
*Penicillium italicum* (OR770486)	0	62.75	68.75
*Stemphylium vesicarium* (OR770487)	0	20	20

**Table 3:** The percentage (%) of spoiled tomato fruit after 2, 4, and 7 days of being inoculated with different pathogens strains.

Fungi	Days		
	2	4	7
*Botrytis cinerea*	0	68	100
*Rhodotorula mucilaginosa*	16	64	100
*Penicillium expansum*	55	100	-
*Alternaria alternata*	20	96	100
*Penicillium italicum*	55	100	-
*Stemphylium vesicarium*	0	60	100

## Data Availability

The datasets presented during this study are available from the corresponding author on reasonable request.
